# Cystathionine beta synthase deficiency and brain edema associated with methionine excess under betaine supplementation: Four new cases and a review of the evidence

**DOI:** 10.1002/jmd2.12092

**Published:** 2020-01-08

**Authors:** Bernd C. Schwahn, Thomas Scheffner, Hedwig Stepman, Peter Verloo, Anibh M Das, Janice Fletcher, Henk J Blom, Jean‐Francois Benoist, Bruce A. Barshop, Jaime J. Barea, Annette Feigenbaum

**Affiliations:** ^1^ Willink Metabolic Unit, Manchester Centre for Genomic Medicine Manchester University Hospitals NHS Foundation Trust Manchester UK; ^2^ Klinikum am Steinenberg, Klinik für Kinder und Jugendmedizin School of Medicine University of Tübingen Reutlingen Germany; ^3^ Laboratory for Metabolic diseases Ghent University Hospital Ghent Belgium; ^4^ Department of Pediatric Neurology and Metabolic Diseases University Hospital Ghent Ghent Belgium; ^5^ Medizinische Hochschule Hannover Klinik für Pädiatrische Nieren‐, Leber‐ und Stoffwechselerkrankungen Hannover Germany; ^6^ Genetics and Molecular Pathology SA Pathology Adelaide Australia; ^7^ Metabolic Unit, Department of Clinical Genetics Center for Lysosomal and Metabolic Diseases. Erasmus Medical Center Rotterdam The Netherlands; ^8^ Service de Biochimie Hormonologie Hôpital Robert Debré, APHP Paris France; ^9^ Department of Pediatrics, Division of Biochemical Genetics, Rady Children's Hospital‐San Diego University of California San Diego California

**Keywords:** adverse drug effect, betaine, brain edema, CBS deficiency, encephalopathy, homocystinuria

## Abstract

CBS deficient individuals undergoing betaine supplementation without sufficient dietary methionine restriction can develop severe hypermethioninemia and brain edema. Brain edema has also been observed in individuals with severe hypermethioninemia without concomitant betaine supplementation. We systematically evaluated reports from 11 published and 4 unpublished patients with CBS deficiency and from additional four cases of encephalopathy in association with elevated methionine. We conclude that, while betaine supplementation does greatly exacerbate methionine accumulation, the primary agent causing brain edema is methionine rather than betaine. Clinical signs of increased intracranial pressure have not been seen in patients with plasma methionine levels below 559 μmol/L but occurred in one patient whose levels did not knowingly exceed 972 μmol/L at the time of manifestation. While levels below 500 μmol/L can be deemed safe it appears that brain edema can develop with plasma methionine levels close to 1000 μmol/L. Patients with CBS deficiency on betaine supplementation need to be regularly monitored for concordance with their dietary plan and for plasma methionine concentrations. Recurrent methionine levels above 500 μmol/L should alert clinicians to check for clinical signs and symptoms of brain edema and review dietary methionine intake. Levels approaching 1000 μmol/L do increase the risk of complications and levels exceeding 1000 μmol/L, despite best dietetic efforts, should be acutely addressed by reducing the prescribed betaine dose.

1

**SYNOPSIS**

Hypermethioninemia can cause acute encephalopathy and patients with CBS deficiency on betaine supplementation without sufficient methionine restriction are at particular risk of the rare complication of brain edema, once plasma methionine levels approach 1000 μmol/L.

## 2 INTRODUCTION

Methionine is an essential amino acid for humans. Excessive intake, however, causes methionine accumulation.^1^, ^2^ Hypermethioninemia without dietary excess is also observed in rare genetic defects of methionine utilization^3^, ^4^, ^5^ or catabolism.^2^ Acute severe hypermethioninemia can be associated with adverse neurological outcomes, including death.

Betaine (trimethylglycine) is a product of choline catabolism and has a role as intracellular organic osmolyte and methyl donor for the remethylation of homocysteine to methionine. Betaine supplementation leads to a decrease of homocysteine and increase in methionine and has been used for over 40 years in the treatment of genetic defects of folate‐dependent remethylation.^6^, ^7^ While dietary methionine restriction is sufficient to treat homocystinuria due to cystathionine beta‐synthase deficiency (CBS deficiency, OMIM # 236200) dietary goals are difficult to achieve and betaine is often used as adjunctive treatment.^8^, ^9^, ^10^

Yaghmai et al^11^ first reported acute symptomatic brain edema in a 10‐year‐old girl with CBS deficiency who had developed plasma methionine concentrations of 2272‐3037 μmol/L after a few weeks of taking betaine more consistently, without adequate dietary methionine restriction. The edema was reversible when betaine was discontinued and strict dietary control instated. Since then, another five similar cases were published 12, 13, 14, 15, 16 as well as a case of brain edema in a child with methionine adenosyltransferase (MAT) type I/III deficiency (OMIM # 250850) on accidental betaine supplementation.^17^ While there is a causal relationship between betaine supplementation and hypermethioninemia in disorders affecting the catabolism of methionine, it is not clear whether brain edema is caused by methionine accumulation or whether increased betaine concentrations are a contributing factor.

A survey of treatment practices for patients with CBS deficiency across Europe identified a large proportion of patients who are on a betaine supplement and 34% of those apparently without dietary methionine restriction.^18^ The published cases and additional reports of adverse outcomes of patients on betaine supplementation to the license‐holder for medicinal betaine under their post‐marketing authorization safety surveillance program raised concerns about the safety of betaine treatment in CBS deficiency and prompted a review of the incidence of brain edema in this population.

## 3 METHODS

We performed a literature search to identify patients who experienced clinical or brain imaging signs of methionine neurotoxicity, in particular of acute brain edema, with or without betaine supplementation.

In addition, we approached clinical metabolic specialists who participate in a large international email‐based discussion forum (Metab‐L, A mailing‐list on Inborn Errors of Metabolism, http://www.metab-l.de) and the sponsor of the European post‐authorization safety study for betaine^19^ to identify potential further unpublished cases. This project was endorsed by the European network for Homocystinurias and Remethylation Disorders (E‐HOD).

Using a standardized questionnaire, we extracted data from published articles and from case notes of unpublished patients with consent from patients or carers, apart from historic case A2 where this was impossible. Data were anonymized for analysis.

## 4 RESULTS

A total of 19 cases were identified who, on the background of hypermethioninemia, either displayed clinical signs of acute brain edema or who underwent cerebral imaging and were found to have white matter changes compatible with edema. These were 15 individuals with CBS deficiency, 1 with MAT I/III deficiency and three healthy individuals without a disorder of methionine or homocysteine metabolism. Appendix 1 contains short narratives of all patients that could be identified for the period from 1997 until 2016, that is, over 20 years.

An overview of the patients' clinical characteristics is provided in Table 1. Relevant dietary and biochemical data are shown in Table 2 and references are given in Table 3.

**Table 1:** Clinical characteristics at diagnosis and around the episode of increased intracranial pressure of all 19 patients

		Diagnosis and treatment				Acute episode of raised ICP or of white matter changes			
Pat ID	Sex	Diagnosis	Symptoms at Diagnosis	Age when diet started	Age when betaine started	Onset	Latency from rise in Met	Other diagnoses	Outcome
A1	F	CBS	NBS	1 wk	1 y, strict from 10 y 1 mo	10 y 6 mo	8‐10 wk	P, ID	FR
A2	F	CBS	Lens subluxation, tall stature	6 y 6 mo	6 y 10 mo	14 y 7 mo	7 y 9 mo	P	Died
A3	F	CBS	Lens subluxation, DD	3 y 2 mo	3 y 3 mo	3 y 5 mo	2‐4 wk	URTI, ID	FR
A4	M	CBS	Lens subluxation, dyspraxia	4 y 6 mo	5 y	5 y 6 wk	2‐4 wk	Nil	FR
A5	F	CBS	Lens subluxation, ID	6 y 9 mo	6 y 10 mo, increased from 6 y 11 mo	7 y 4 mo	16‐18 wk	ID	FR
A6	F	CBS	NBS	1 mo	Early childhood, irregular	24 y	6‐8 wk	P, S, ID	FR
A7	M	CBS	SSS thrombosis	4 y 3 wk	4 y 4 wk	4 y 4 mo	10‐12 wk	SSS remnant	FR
A8	M	CBS	NBS	1 wk	2 y, poor compliance from 19 y	21 y	1‐2 y	Nil	FR
A9	M	CBS	NBS	1 mo	6 mo, poor compliance	2 y 5 mo	>20 wk	Nil	FR
A10	M	CBS	SSS thrombosis and ID	2 y 1 mo	2 y 3 mo	2 y 5 mo	6‐8 wk	Nil	FR
A11	M	MAT I/III	NBS, assumed CBS def	3 wk	3 wk	3 y 10 mo	1‐2 y	Nil	FR
B1	F	Healthy	N/A	N/A	N/A	69 y	2 h	AH	Died
B2	M	Healthy	Repeat NBS	N/A	N/A	4 mo (corr 1 mo)	6 wk	Premature 28w	FR
B3	F	Healthy	MRI brain in critical care	N/A	N/A	2 wk	1‐2 wk	PPHN, TPN	FR
C1	M	CBS	Stroke	9 y	2d after diagnosis	9 y	N/A	Stroke	N/A
C2	M	CBS	ID, left sided weakness	3 y	N/A	3 y, on Dx	N/A	ID	FR
C3	M	CBS	Lens subluxation, ID	7 y	7 y, poor compliance	9 y	N/A	S, ID	N/A
C4	F	CBS	Lens subluxation, ID	6 y	6 y, poor compliance	7 y	Headaches 7 y	ID	FR
C5	F	CBS	DD, Myopia	3 y 10 mo	4 y, poor compliance	6 y	N/A	ID	N/A

Abbreviations: ICP, intracranial pressure; ID, intellectual disability; S, spasticity of limbs; SSS, superior sagittal sinus; P, pancreatitis; URTI, upper respiratory tract infection; PPHN, persistent pulmonary hypertension of the newborn; TPN, total parenteral nutrition; AH, arterial hypertension; NBS, General newborn screening programme; FR, full recovery.

**Table 2:** Protein and betaine intake and blood metabolites during the acute episode of brain edema

Pat ID	Diagnosis	Protein or Met intake Fold of MSI^20^	Betaine dose (g/day)	Betaine dose (mg kg^−1^ day^−1^)	Met at presentation (μmol/L)	tHcy at presentation (μmol/L)
A1	CBS	2.25	6	200	2272‐3037	263‐278
A2	CBS	Inconsistent Met restriction	4.5	N/A	1857‐3154	N/A
A3	CBS	N/A	6	400	1207	260
A4	CBS	1.4	3	150	1190‐1205	239
A5	CBS	1.2	4.5	200	1022‐1125	116‐143
A6	CBS	Inconsistent Met restriction	1	N/A	559‐1282	266
A7	CBS	N/A	6	240	1285	N/A
A8	CBS	>2	7.5	N/A	678‐1142	167
A9	CBS	0.75	4.1	340	1182‐1211	148‐203
A10	CBS	N/A	2	107	738‐972	120
A11	MAT I/III	Inconsistent Met restriction	Unknown	N/A	960‐1560	6‐15
B1	Healthy	60‐80 (single dose of 80 g Met)	Nil	Nil	4640‐5760	25‐43
B2	Healthy	4 (276 mg/kg per day from 2 mo 3wk)	Nil	Nil	2326‐6830	44
B3	Healthy	10 (507 mg/kg per d)	Nil	Nil	1300‐2154	27
C1	CBS	Unrestricted diet	6	N/A	433 (10d after adm)	N/A
C2	CBS	Inconsistent Met restriction	Unknown	N/A	1082	175
C3	CBS	Inconsistent Met restriction	5	N/A	495 at dx	154 at dx
C4	CBS	Inconsistent Met restriction	Unknown	N/A	622 at dx	173 at dx
C5	CBS	Inconsistent Met restriction	8	N/A	N/A	N/A

Abbreviations: MSI, minimum safe intake; tHcy, plasma total homocysteine; Met, plasma methionine.

**Table 3:** References for patient data

Pat ID	Diagnosis	Reference
A1	CBS	11
A2	CBS	J. Fletcher, personal communication
A3	CBS	A. Das, personal communication
A4	CBS	12
A5	CBS	T. Scheffner, personal communication
A6	CBS	16
A7	CBS	13
A8	CBS	15
A9	CBS	14
A10	CBS	H Stepman and P Verloo, personal communication
A11	MAT I/III	17
B1	Healthy	1
B2	Healthy	21
B3	Healthy	21
C1	CBS	19
C2	CBS	22
C3	CBS	23
C4	CBS	23
C5	CBS	23

Cases were stratified into three separate groups:

### 4.1 Group A

A total of 11 patients with CBS deficiency (Cases A1‐A10) or MAT I/III deficiency (A11) suffered from acute brain edema in association with hypermethioninemia and betaine supplementation. Five of those cases (A2, A3, A5, A9, A10) have not or only partly been published before.

The patients' metabolic diagnosis was based on either general newborn screening programs or following onset of clinical symptoms between the ages of 2 and 6.75 years. They were started on pyridoxine supplementation and a methionine restricted diet soon after. Supplementary betaine was introduced usually a few months after implementation of dietary and cofactor treatment, apart from case A11 where it was given from the time of diagnosis. Brain edema occurred between 6 weeks and 20 years after starting betaine. The latency between suspected or documented persistent severe hypermethioninemia and the onset of brain edema was shorter: 2 weeks to 7 years 9 months, with a median of 9 weeks.

Immediately before and around the time of presentation, the lowest individual plasma methionine concentrations ranged from 559 to 2272 μmol/L (median 1182) and the highest from 972 to 3154 μmol/L (median 1211). Plasma concentrations of betaine and its metabolite dimethylglycine (DMG) on the day of admission to hospital were available for three cases, A4, A5, and A9: Betaine was 98, 131, and 40 μmol/L and DMG was 64, 43, and 24 μmol/L, respectively. All were within the expected trough range under treatment.^6^ CSF concentrations were available for two cases: in patient A4 methionine was 235 and betaine 6.6 μmol/L; in patient A9 methionine was 199, betaine 0.12 and DMG 1.55 μmol/L.

Treatment of brain edema consisted of unspecific measures to lower intracranial pressure and of tighter dietary restriction of methionine. Betaine was temporarily stopped in seven patients (A1, A3, A4, A5, A8, A10, A11) and the dose halved in two patients (A6, A9). The other two patients (A2, A7) remained on an unchanged dose while being treated for edema. Betaine was later re‐introduced in four of the seven temporarily discontinued patients (A3, A5, A8, and A10). There was no recurrence of brain edema in the seven surviving patients who continued on long term betaine supplementation. Betaine doses were not always detailed, known doses ranged from 100 to 400 mg kg^−1^ day^−1^ or 1.0 to 7.5 g day^−1^ (Table 2).

All patients except A2 survived and completely recovered from brain edema. Patient A2 was admitted with acute severe pancreatitis but also displayed abnormal movements and was found to have the hypodense white matter on CT brain scan. She died from multiorgan failure. Post mortem examination demonstrated increased brain weight and spongiosis. The contribution of brain edema to the fatal outcome is unclear.

### 4.2 Group B

Acute brain edema was observed in two healthy infants at the age of 2 weeks and 4 months, respectively, who had been on an inappropriately high oral intake of protein from formula feeds.^21^ They were diagnosed accidentally after a respective exposure of 1 and 6 weeks long to methionine plasma concentrations of 1300‐6830 μmol/L. Both recovered on adjusting their feeds.

The third healthy individual who experienced acute methionine neurotoxicity was reported by Cottington et al.^1^ Patient B1, a healthy woman of 69 years, deteriorated with signs of encephalopathy within 2 hours of a methionine loading test. Her plasma methionine reached concentrations of up to 5760 μmol/L. The authors suspected an accidental 10‐fold methionine overdose as the most likely reason for her excessive hypermethioninemia. She died after a prolonged and complicated comatose state.

### 4.3 Group C

Further five cases have been reported of patients with longstanding hypermethioninemia due to CBS deficiency and various degrees of cerebral white matter signal changes compatible with edema.

Case 1 (C1) developed acute brain edema due to cerebral venous thrombosis prior to diagnosis of CBS deficiency and before betaine was administrated [unpublished]. Case 2 (C2) had white matter signal changes that resolved on initiation of dietary treatment and betaine supplementation.^22^ Three other patients on dietary and medical treatment, including betaine, but with apparently inconsistent adherence to treatment (C3, C4, and C5) were found to have white matter changes on imaging. In one patient (C4) these were associated with clinical signs compatible with raised intracranial pressure and resolved with time.^23^ Unfortunately, the case reports for patients C3, C4, and C5 lack further clinical and biochemical detail.

In Group C, patients had maximum documented plasma concentrations of methionine ranging from 433 to 1082 and of total homocysteine from 154 to 175 μmol/L.

Betaine doses in this group were not always given in detail; known doses ranged from 5 to 8 g day^−1^.

## 5 DISCUSSION

The acute and long‐term toxicity of methionine excess has been studied for decades.^24^ When acute brain edema in a patient with CBS deficiency and high methionine concentrations under betaine supplementation was first described, the role of betaine as an organic osmolyte raised suspicion that an excess of betaine rather than of methionine may cause edema. Betaine, however, has low toxicity and has been administered in high doses, up to 20 g per day, in patients with homocysteine remethylation defects without adverse effects. After oral dosing betaine has a short distribution half‐life of 0.6 hours.^6^, ^25^ Plasma concentrations vary from normal (20‐60 μmol/L) to above 2800 μmol/L during typical dosing.^6^, ^26^, ^27^ Plasma betaine concentrations are not routinely monitored. We were able to retrieve data from patients A4, A5, and A9 whose plasma betaine concentrations levels were not elevated at the time of presentation with brain edema. The available concentrations of betaine and DMG in CSF were very low and do not indicate any accumulation of betaine. Animal studies suggest that brain tissue stores only small amounts of betaine.^28^, ^29^ The betaine transporter BGT‐1 is expressed in leptomeninges but is only poorly expressed in neuronal or glial cells and a recent review concluded that the contribution of betaine to maintaining brain cellular volume is probably insignificant.^30^

Amino acid transport across brain diffusion barriers involves saturable carriers and non‐saturable diffusion (Smith et al. 1987). Methionine in very high concentrations can displace other neutral amino acids from high‐affinity carriers (reviewed by Chien et al.^4^) causing preferential uptake and a relative lack of other essential substrates in the brain. Furthermore, at very high plasma concentrations significant amounts of methionine may enter the brain cells via low‐affinity carriers^32^ or by simple diffusion. CSF concentrations of methionine are normally below 10 μmol/L but reached over 20‐fold higher concentrations in patients A4 and A9. Short term increases in plasma methionine concentrations to a range of 700‐1800 μmol/L, such as seen during a typical L‐methionine loading test, frequently lead to mild encephalopathy manifesting as dizziness, sleepiness, and nausea.^33^, ^34^ Methionine‐induced increased oxidative stress and inhibition of synaptic Na+,K+‐ATPase, which is required to regulate neuronal cell volume, has been suggested by a number of authors^11^, ^21^, ^35^ as the most plausible explanation for the development of brain edema.

Our search identified further cases of brain edema in patients with hypermethioninemia under betaine supplementation (group A). However, brain edema also occurred in patients with severe hypermethioninemia without concomitant betaine treatment (group B). A few patients (group C) were included who demonstrated signs of white matter disease after long‐term exposure to increased methionine concentrations, indicating the possibility of chronic methionine neurotoxicity. White matter lesions in CBS deficiency can represent both dysmyelination and intramyelinic edema. The reversibility of such lesions in cases C2 and C4 and as reported by Franco et al^36^ suggests the presence of edema.

These observations lead to the conclusion that methionine is the primary toxic agent causing brain edema, and not betaine. Even mildly impaired CBS activity reduces the capacity to metabolize methionine.^34^ Without adequate methionine restriction betaine supplementation greatly exacerbates methionine accumulation in patients with CBS deficiency and becomes a permissive factor for the development of brain edema.

We were able to identify only 16 cases of manifest brain edema in patients with CBS deficiency over a period of 20 years. The relative rarity of this complication despite a considerable prevalence of severe hypermethioninemia indicates a low relative risk and additional precipitating factors. Most episodes of edema occurred shortly after starting or increasing the betaine supplement, which suggests that acute changes are more dangerous than chronic hypermethioninemia. On the other hand, not all patients showed clear clinical symptoms and some cases may have been overlooked. It is possible that a higher susceptibility to edema is conferred by variations in cerebral methionine transport or synaptic Na+,K+‐ATPase in some individuals but this has not been tested.

In the reported cases, methionine plasma concentrations were not always frequently enough recorded to allow firm conclusions about a threshold above which edema can develop. Around the time of manifestation, none of the individuals with brain edema had a plasma methionine lower than 559 μmol/L. Edema was however observed in one patient whose recorded maximum level was 972 μmol/L. While levels below 500 μmol/L can be deemed safe, it appears that brain edema can develop with plasma methionine concentrations below 1000 μmol/L. It is not safe to aim for plasma levels below 1500 μmol/L as suggested previously^11^ but rather below 1000 μmol/L, in accordance with recently published guidance.^9^

## 6 RECOMMENDATION

Patients with CBS deficiency on betaine supplementation need to be regularly monitored for concordance with their dietary plan and for plasma methionine concentrations. Recurrent levels above 500 μmol/L should alert clinicians to check for clinical signs and symptoms of brain edema and review the dietary methionine intake. Levels approaching 1000 μmol/L do increase the risk of complications and levels exceeding 1000 μmol/L, despite the best dietetic efforts, should be acutely addressed by reducing the prescribed betaine dose.

## CONFLICT OF INTEREST

B Schwahn reports personal fees from Orphan Europe, outside the submitted work. Other authors do not report any conflict of interest. T.S., H.S., P.V., A.D., J.F., H.J.B., J.F.B., B.A.B., J.J.B., and A.F. declare they have no conflict of interest.

## PATIENT CONSENT STATEMENT

Consent from patients or carers for publication of anonymized data were obtained by the treating physicians.

## AUTHOR CONTRIBUTIONS

BCS coordinated the process of data collection and analysis, drafted the manuscript. HJB and JFB supported the conception and design of this work. T.S., H.S., P.V., A.D., J.F., B.A.B., J.J.B., and A.F. obtained patients' consent and provided anonymized data from their respective patients. All authors critically reviewed the manuscript and contributed to the final version.

## Supporting information


**Appendix S1**: Supporting Information
