# Supplementary material for: Cystathionine beta synthase deficiency and brain edema associated with methionine excess under betaine supplementation: Four new cases and a review of the evidence
Source: JIMD Rep. 2020 Jan 8;52(1):3–10. doi: 10.1002/jmd2.12092 (PMC7052692; doi:10.1002/jmd2.12092)
Supplement: Supplementary file 1 — Appendix S1: Supporting Information [file JMD2-52-3-s001.docx]

*Supplementary Case narratives*

A1 [Yaghmai et al 2002]. A child had been diagnosed with CBS deficiency by general Newborn Screening and had been on a methionine-restricted diet and betaine for many years but with inconsistent adherence. Treatment was interrupted when she developed splenic vein thrombosis and acute pancreatitis at the age of 10 years. Treatment was more consistently re-instated after recovery. However, while betaine was consistently administered methionine restriction was not maintained and the patient developed severe hypermethioninaemia. Signs of increased intracranial pressure occurred within 8-10 weeks and brain edema was confirmed by MRI. There was no intracerebral thrombosis. Betaine was discontinued and a strict methionine restriction was enforced. Plasma methionine concentrations subsequently decreased and the patient recovered fully. Betaine was not re-started. The patient again lapsed into poor dietary control and methionine concentrations were elevated, albeit not above 1000μmol. There was no recurrence of brain edema.

A2 [J Fletcher]. A 6 year old girl was diagnosed with CBS deficiency after presenting with lens dislocation, high arched palate and tall stature. Free homocystine was present in urine and plasma. Plasma methionine was 512 and 961μmol/l, respectively, on two occasions. Betaine was introduced at the age of 6 years and 10 months at a dose of 3g twice daily. This abolished the free homocystine but methionine increased to 1203 and 1194μmol/l. The daily betaine dose was reduced to 4.5g, however plasma methionine remained above 1000μmol/l, occasionally reaching 2500μmol/l, over the subsequent 7 years and the patient was poorly compliant to dietary methionine restriction. She developed ulcerative colitis which required subtotal colectomy at the age of 14 years despite immunosuppressive treatment with 5-acetylsalicylate, prednisolone and cyclosporine. The patient was admitted to hospital at the age of 14y 8m with a history of Parkinsonian features over the preceding weeks and with signs of acute pancreatitis and encephalopathy. Plasma methionine after admission was 3154μmol/l and a cranial CT scan demonstrated white matter hypodensity. She was commenced on parenteral nutrition. Methionine decreased to 1857μmol/l over three days. The patient deteriorated further and died shortly after. The autopsy confirmed acute pancreatitis and showed increased brain weight without evidence for thrombosis and florid spongiform changes involving cerebral white matter.

A3 [A Das]. The girl was born at term after an uncomplicated pregnancy. Psychomotor development was delayed. Genua valga were noticed when she started walking. An ophthalmological exam revealed subluxation of the lenses at the age of 3 years. At 3 years and 2months of age plasma total homocysteine was 278μmol/l and methionine 227μmol/l (normal 7-40). She was diagnosed with pyridoxine non-responsive CBS deficiency and started on a methionine restricted diet and betaine 3 x 2g/day (400 mg/kg per day). Two months after initiation of betaine, the girl suffered from headache, recurrent vomiting (not related to meals) after an episode of febrile upper respiratory tract infection. Palsy of N. abducens developed. Ophthalmological work-up revealed papilledema and an MRI revealed brain edema. Steroids were given for 4 days accompanied by diuretics to symptomatically treat brain edema. Medication was stopped and the girl recovered over a period of 7 days. Methionine reached a peak concentration of 1207μmol/l during acute illness and fell to 400-500μmol/l later on after recovery. Homocysteine levels were elevated up to 260μmol/l during the episode of brain edema. The girl recovered without clinical sequelae, betaine was restarted at a dose of 3 x 1g without clinical complications over the course of 20 years.

A4 [Devlin et al 2004]. A 4.5 year-old male patient was diagnosed with CBS deficiency after presenting with dislocated lenses and motor dyspraxia. Baseline tHcy was 334-430, plasma methionine 203. The patient started on a methionine restricted diet and oral betaine at the age of 5 years. Four weeks later the patient experienced morning headaches and vomiting, which increased in frequency and severity. Cranial MRI and angiography later showed no thrombosis but widespread abnormal signal in the hemispheric white matter. Lumbar puncture revealed an opening pressure of 47cm CSF. Plasma Methionine was 1205uM. Betaine treatment was doubled to 3 g twice daily. Under conservative management and four days after the increase in betaine, symptoms worsened. The opening pressure increased to 80 cm CSF. Methionine in plasma was 1190μmol/l (normal range 10-54). tHcy was 239μmol/l. Betaine was 98 and DMG 64μmol/l. CSF methionine was 235 and CSF betaine 6.6μmol/l. The patient underwent intubation/ventilation, and received mannitol as corrective treatment. Bilateral frontotemporal decompressive craniotomies were performed. CT showed diffuse brain swelling and invasive angiography ruled out thrombosis. Betaine treatment was discontinued. Pupillary responses normalized within 6 hours of surgery. Ventilation and fluid restriction were continued for 48 hours, after which the patient recovered consciousness. Neurological examination was normal at discharge 8 days later and at further follow-up. After 6 months, a cranial MRI showed resolution of the oedema and the white matter signal abnormalities. The patient was continued on a methionine restricted diet with good metabolic control. Betaine was never restarted.

A5 [T Scheffner]. The patient was diagnosed at the age of 6 years and 9 months due to lens dislocation and severe myopia, and impaired psychomotor development. Baseline tHcy was 257-274 and methionine 39μmol/l. Treatment with a methionine restricted diet was initiated but poorly accepted. Betaine was introduced after four weeks at a dose of 0.7g three times daily (100mg/kg) and increased after two months to 1.5 g three times a day when tHcy was still increased to 143μmol/l. Due to dietary non-adherence plasma tHcy remained elevated ranging from 116 to 143μmol/l and methionine was between 1022 and 1125μmol/l. After four months of betaine supplementation the patient started experiencing recurrent headaches with subsequent visual disturbance and lethargy and the family discontinued both the betaine supplement and methionine restriction. 10 days after onset of symptoms, she was admitted with bilateral Nervus abducens paresis and ataxia. Her CT brain showed supratentorial brain swelling and edema. MRI brain ruled out intracranial thrombosis. Plasma tHcy on admission was 134 and Met was 1030μmol/l. Betaine in plasma was 131 μmol/l and DMG was 43 μmol/l (courtesy Dr M Laryea, Duesseldorf, Germany). Betaine supplementation and methionine-restriction were re-instated but betaine was discontinued after one day, upon suspicion of a betaine-related edema. The patient did, however, not improve. Plasma methionine had increased to 1509 and tHcy decreased to 66μmol/l six days into the admission. Subsequent MRIs showed progressive edema. The patient eventually improved after a 5-day course of high-dose methylprednisolone and made a full neurological recovery within 6 weeks. Betaine and DMG concentrations in plasma had normalised within two weeks. On continued methionine restriction tHcy ranged from 66 to 253μmol/l and methionine from 96 to 405μmol/l. A repeat MRI scan 5 months after the episode showed a normal brain. Betaine was reintroduced at a lower dose of 900mg per day (36mg/kg per day) and well tolerated throughout subsequent years.

A6 [Vatanavicharn et al 2008]. A girl who had been diagnosed with CBS deficiency by newborn screening and had been started on dietary treatment and betaine with inconsistent adherence throughout childhood presented with recurrent pancreatitis from the age of 14 years. At 24 years of age another episode of pancreatitis required prolonged total parenteral nutrition for 2 months. Plasma amino acids during this period showed methionine concentrations from 559 to 718μmol/l (normal 14–41). She subsequently developed stupor, tremor of the upper extremities, spasticity, and Babinski responses of the bilateral lower extremities. Two months after the onset of acute neurological deterioration, a brain MRI demonstrated brain edema. Plasma amino acids showed a markedly elevated methionine of 1282μmol/l and total homocysteine (tHcy) of 266μmol/l (normal 4–10). Methionine restriction was intensified and betaine was continued at a dose of 1g/day. Methionine level decreased to 587μmol/l; tHcy decreased to 59μmol/l. Four weeks later, she returned to full consciousness, and her lower extremities became less spastic. Methionine had by then decreased to a low normal level of 31μmol/l. Betaine dosage was maximized to 7g/day. She remained neurologically stable even though she never returned to her previous status, and was wheelchair dependent. She was still noncompliant with treatment. At 29 years of age, she again developed pancreatitis and a deep vein thrombosis of the upper extremity, and became agitated and disoriented. A brain MRI at this time was normal. Plasma tHcy at that time was 129μmol/l and methionine was 998μmol/l.

A7 [Ismayilova et al 2018]. A 6-year-old boy with learning disability and marfanoid features was diagnosed with homocystinuria with initial total homocysteine of 344μmol/l and methionine 64μmol/l. His brain MRI scan was normal. At age 6.5 years he developed superior sagittal sinus (SSS) thrombosis. He was started on warfarin and treated with a low methionine diet, betaine, folic acid and pyridoxine. On a dose of 2g betaine three times daily (240mg/kg per day) and due to poor dietary adherence plasma methionine levels continued to rise (1285μmol/l). He developed left-sided weakness. A MRI brain scan 3 months later showed new confluent widespread T2 hyperintensity in previously myelinated white matter while the SSS had started to recanalize. With 3-month further treatment of tight dietary control, his methionine fell to 233μmol, his neurological deficit resolved and another MRI scan confirmed complete resolution of white matter changes and SSS recanalization..

A8 [Sasai et al 2015]. A child was diagnosed by newborn screening with pyridoxine non-responsive CBS deficiency and treated with a methionine-restricted diet and from the age of 2 years with betaine. Normal neurological development was achieved due to good adherence to treatment until age 19 years, with a dose of 5g betaine per day. Living independently his dietary control lapsed with an estimated intake of 1000-2000mg Methionine per day. Betaine was increased to 3.75 g twice daily after 1 year. His dietary control did however not improve and he had persistently high plasma methionine concentrations of 678 to 1142μmol/l. At 21 years of age he initiated aspirin to prevent cerebral infarction, and at the same time complained of mild headaches. Brain MRI and magnetic resonance angiography (MRA) showed no evidence of vascular obstruction or stenosis. However, T2-weighted images (T2WI) and fluid attenuated inversion-recovery (FLAIR) images showed diffuse high signal intensity in subcortical areas extending to the deep white matter. On admission his neurological examination was unremarkable and he had a plasma methionine of 904μmol/l and tHcy of 167μmol/l. Lumbar puncture showed an opening pressure of 18 cm cerebrospinal fluid (CSF), but was otherwise normal. CSF methionine and tHcy were 116μmol/l (normal range 1-5) and 1.4μmol/l (normal range 0.04-0.13) respectively. Betaine was stopped. 3 weeks later, the patient was hospitalised and started on a methionine-restricted diet. His headache frequency decreased gradually after methionine restriction. Plasma methionine decreased to 147μmol/l and tHcy increased to 263μmol/l on the 8th hospital day. Thus, oral betaine supplementation was restarted carefully at 5 g/day on the 13th hospital day. Thereafter, plasma concentrations of methionine and tHcy were stable, at 190 and 137μmol/l, respectively, on the 18th hospital day. He was discharged home after 30 days and protein restriction and oral betaine therapy were continued with good compliance. Serial MRIs showed mild edema prior to admission and pronounced edematous changes during the acute admission that resolved over the course of 5 months with strict dietary treatment.

A9 [Li et al 2018]. A girl was identified with CBS deficiency by NBS. The diagnosis was confirmed at the age of 4 weeks when she had a tHcy of 307 and methionine of 828μmol/l. She was commenced on a low methionine diet and betaine was started at 6 months of age with a dose of 100mg/kg per day and then increased. By the age of 2 years she was prescribed up to 14g total protein per day (approximately 280mg methionine or 20mg/kg) but she was not taking her methionine free amino acid supplement regularly. Betaine was given regularly at a dose of 5g/day (370mg per kg in three doses) and she had levels of tHcy of 110-156 and of methionine of 1007-1211μmol/l. At the age of 2y 5m she had an episode of viral illness which started 10 days prior to admission and experienced transient ataxia. She had a routine eye examination shortly after, which revealed papilledema. On admission to hospital she had a plasma tHcy of 203, methionine of 1182μmol/l, betaine of 39.7 and DMG of 24.0μmol/l. A brain MRI scan revealed extensive supratentorial, brainstem and cerebellar cytotoxic edema, with restricted diffusion. The CSF opening pressure was 28cm CSF. Methionine in CSF was 199, betaine was 0.12 and DMG 1.55μmol/l. Dietary protein was withheld. After one day tHcy was 189 and Methionine 724umol/l. Betaine was continued but the dose decreased to 2.2g/day (150mg/kg per day) in 2 doses. Ensuring better adherence to the methionine-restricted diet tHcy had decreased to 122 and methionine to 207umol/l, respectively, two weeks later. There was no follow-up MRI reported.

A10 [H Stepman and P Verloo]. A boy with global cognitive developmental disorder was diagnosed at 2 years 1month of age with sinus venous thrombosis. Further investigations revealed a tHcy of 264 and a methionine of 459umol/l in plasma and a diagnosis of CBS deficiency was made. He was started on a methionine restricted diet and pyridoxine. Betaine supplementation was started at 2y and 3m of age with a dose of 2g per day in 2 doses (107mg/kg per day) and taken regularly, resulting in a tHcy of 120 and a methionine of 738umol/l a few weeks later. At 2y 5m of age he manifested with headaches and vomiting. One week into his symptoms a MRI head showed diffuse signal abnormalities in white matter and in the globi pallidi with diffusion restriction in all white matter. A lumbar puncture was performed and showed a CSF methionine of 96μmol/l (ref 0-6.7). The opening pressure was not recorded. Betaine was discontinued and serial daily bloods showed increasing tHcy of 115, 176, 186, 180μmol/l and decreasing plasma methionine concentrations of 972, 582, 340, 350μmol/l. He recovered over the course of 2 weeks. Pyridoxine was eventually stopped and betaine reintroduced after 2 months, at the previous dose of 2g/day and was well tolerated. Dietary methionine restriction was tightened, including an amino acid supplement. The child remained asymptomatic over the next 6 months with concentrations of tHcy ranging from 2 – 129μmol/l and of methionine from 9 – 1012μmol/l.

A11 [Tada et al 2004]. A currently 5-year-old boy was found to have pronounced hypermethioninemia on neonatal screening. In addition to elevated plasma methionine (740 μmol/l; normal, 40), laboratory studies showed elevated total homocysteine (37μmol/l; normal range, 3.0 –14.0), leading to a diagnosis of CBS deficiency. At the age of 3 weeks, he began treatment with methionine-restriction and oral betaine; pyridoxine was started at age 2.5 years because of difficulty in maintaining an adequately restrictive diet. His early development was normal, and his neurologic examination was unremarkable. During the treatment, plasma methionine was markedly elevated (960– 1560μmol/l), but total homocysteine (6.3–14.9μmol/l) and other plasma amino acids were normal. After pyridoxine administration, he started to have mildly decreased appetite and sleepiness in the daytime. Neurologic examination was unremarkable. The results of routine laboratory studies on blood and urine were either within normal limits or negative. MR imaging was performed at the age of 3 years 10 months to evaluate for possible cerebral lesions. MR imaging showed abnormal T1 and T2 signals and reduced diffusion in the cerebral white matter. Findings were suggestive of MAT I/III deficiency rather than CBS deficiency. After discontinuation of all the therapy at 4 years of age, his clinical symptoms disappeared. Plasma concentration of methionine were 570–1090μmol/l and of total homocysteine 14 –22μmol/l. Follow-up MR imaging at 5 years demonstrated normalization of white matter signal intensity.

B1 [Cottington et al 2002]

A 69-year-old woman underwent a standard methionine-loading test for research purposes. She was healthy apart from arterial hypertension. Two hours and 40 min after ingesting a planned dose of 8.39g of L-methionine (100mg/kg body weight and approximately 3-4 times a usual daily intake) she started vomiting and then became increasingly confused and combative and finally comatose. She died 30 days later from complications arising from this episode. Her plasma methionine concentration after the load had risen to 4640μmol/l after 2h and to 5760μmol/l after 4h. Peak concentrations of methionine were at least 3 times higher than expected from the dose given and prompted the authors to investigate and rule out the possibility of an underlying metabolic disorder. They conclude that an accidental 10-fold methionine overdose was the most likely reason for excessive hypermethioninaemia and that cerebral symptoms were suggestive of raised intracranial pressure. The case report discusses the evidence for neurotoxic effects of hypermethioninaemia from other, historical cases.

B2 [Mudd et al 2003]

Mudd and colleagues investigated the etiology of hypermethioninaemia that had been identified in 10 young infants through general newborn screening or nutritional monitoring. They established an inappropriately high nutritional intake as cause but also discovered that two of the infants had signs of cerebral edema in association with hypermethioninaemia. Patient B2 is Case 1 in their case series. This is a preterm baby who was switched to an infant formula with very high methionine content from age 83 days (corrected age term) providing 276mg/kg per day of methionine. Plasma methionine was measured during the following weeks and ranged from 1810 to 6830μmol/l. The child became irritable after 5 weeks and a brain MRI showed diffusely increased T2 signal throughout the supratentorial white matter and within the brain stem, particularly within the inferior colliculi. Early postnatal and repeat scans after normalisation of methionine concentrations showed normal brain.

B3 [Mudd et al 2003].

Patient B3 is Case 10 in the original case series. A term neonate was treated for persistent pulmonary hypertension due meconium aspiration and received parenteral nutrition with an inappropriately high methionine content, providing an intake of up to 507mg/kg per day. Her plasma methionine rose to 1300μmol/l after 9 days and 2154 μmol/l after 13 days. A brain MRI at the same time showed diffusely increased T2 signal throughout the supratentorial white matter and within the brain stem, particularly within the inferior colliculi. Her methionine intake was restricted and plasma methionine normalised within 4 days. A repeat MRI 20 days later showed improvement and after 6m complete normalisation.

C1 [Valayannopoulos et al 2019]. A 9-year-old healthy boy presented with decreased consciousness level and arterial hypertension. Imaging revealed cerebral venous thrombosis presenting with intraparenchymatous hemorrhage for which the patient received heparin, midazolam, barbiturate and endotracheal intubation. On finding hyperhomocysteinaemia and elevated plasma methionine he was subsequently diagnosed with CBS deficiency. 48h after admission the patient was started on oral betaine 6000 mg divided in 2 intakes. The patient was also treated with vitamin B6 and folic acid. One week into his treatment, plasma methionine was 433μmol/l. One day later the patient experienced recurrent cerebral venous thrombosis that caused brain oedema, and fatal intracranial hypertension.

C2 [Brenton et al 2014].

A 4-year-old boy with cognitive developmental disorder and onset of focal neurological signs underwent a cranial MRI and was found to have diffusely increased T2 signal and restricted diffusion throughout the supratentorial white matter and dorsal pontine tracts. At the time his plasma tHcy was 175 and methionine 1082μmol/l. He was diagnosed with CBS deficiency and started on a methionine restricted diet, betaine, folic acid , pyridoxine and aspirine. One year after initiation of treatment the MRI was repeated and showed a resolution of the white matter changes.

C3 [El Bashir H et al 2016]

C4 [El Bashir H et al 2016]

C5 [El Bashir H et al 2016]

The authors report white matter changes in children with untreated CBS deficiency. Three patients (Case 1 - 3 of the original publication are C3, C4 and C5 here) with pyridoxine unresponsive CBS deficiency were found to have chronic leukoencephalopathy under inconsistent treatment, including with betaine. The paper does not provide detailed clinical or biochemical data but the authors state that plasma methionine persistently ranged from 600-1000μmol/l and that decreased methionine concentrations were associated with improving white matter changes in the MRI in two patients.

Patient C3 was diagnosed at the age of 7 years and started on dietary treatment and supplementation with betaine, pyridoxine, folate and hydroxycobalamin but had poor adherence to the diet. At the age of 9 years he was found to have increased tone and exaggerated deep tendon reflexes in his legs. A cranial CT scan showed diffuse hypodensity in his cerebral white matter. His symptoms persisted and a cranial MRI at the age of 13 years showed diffusely increased periventricularT2 signal and restricted diffusion.

Patient C4 was diagnosed at the age of 6 years and started on dietary treatment and supplementation with betaine, pyridoxine, folate and hydroxycobalamin but did not adhere to treatment recommendations. At the age of 7 years she developed recurrent intermittent headaches and a cranial MRI demonstrated a diffusely increased T2 signal in the supratentorial white matter, which persisted in a repeat MRIs. However, a cerebral MRI at the age of 15 years demonstrated complete resolution of the previous white matter abnormalities.

Patient C5 was diagnosed at the age of 3 years and 10 months and started on dietary treatment and supplementation with betaine, pyridoxine, folate and hydroxycobalamin but did not adhere to treatment recommendations. A brain MRI at 5 years of age was reported as normal. A repeat at 8 years of age showed a diffusely increased T2 signal mainly in the periventricular supratentorial white matter, which slowly improved over the course of the following 5 years.
